# Transcranial photobiomodulation (808 nm) attenuates pentylenetetrazole-induced seizures by suppressing hippocampal neuroinflammation, astrogliosis, and microgliosis in peripubertal rats

**DOI:** 10.1117/1.NPh.9.1.015006

**Published:** 2022-03-25

**Authors:** Chung-Min Tsai, Shwu-Fen Chang, Chih-Chuan Li, Hsi Chang

**Affiliations:** a Taipei Medical University, Graduate Institute of Medical Sciences, College of Medicine, Taipei, Taiwan; b MacKay Children’s Hospital, Department of Pediatrics, Taipei, Taiwan; c Taipei Medical University Hospital, Department of Pediatrics, Taipei, Taiwan; d Taipei Medical University, College of Medicine, School of Medicine, Department of Pediatrics, Taipei, Taiwan

**Keywords:** tPBM, photobiomodulation, epilepsy, astrogliosis, microgliosis

## Abstract

**Significance:**

Transcranial photobiomodulation (tPBM) at 808 nm attenuates pentylenetetrazole (PTZ)-induced seizures and convulsive status epilepticus (CSE) in peripubertal rats by protecting neurons from injury and parvalbumin-positive interneurons from apoptosis, and preserving the integrity of perisomatic inhibitory networks. However, the effects of tPBM on neuroinflammation, astrogliosis, and microgliosis in epileptic rat brains are unknown. Thus, further study to unveil these aspects is needed for understanding the phenomena of tPBM on pediatric CSE prevention.

**Aim:**

To evaluate the effects of tPBM on neuroinflammation, astrogliosis, and microgliosis in peripubertal rat hippocampus with PTZ-induced seizures and SE.

**Approach:**

An 808-nm diode laser was applied transcranially to peripubertal rats prior to PTZ injection. Immunofluorescence staining of neuron-specific enolase (NSE) was used as a marker of neuroinflammation, glial fibrillary acid protein (GFAP) for astrogliosis, ionized calcium-binding adapter molecule 1 (Iba-1) for microgliosis, and mitochondrial cytochrome c oxidase subunit 1 (MT-CO1) for confirming the involvement of cytochrome c oxidase (CCO).

**Results:**

tPBM significantly reduced NSE immunoreactivity in CA3 in PTZ-treated rats, GFAP immunoreactivity in CA1, and Iba-1 immunoreactivity in CA3. Enhancement of hippocampal MT-CO1 reflected that tPBM acted in CCO-dependent manner.

**Conclusions:**

tPBM (808) attenuated PTZ-induced seizures and SE by suppressing neuroinflammation, astrogliosis, and microgliosis in peripubertal rats.

## 1 Introduction

Treatment of pediatric epilepsy relies on antiepileptic drugs (AEDs), but the response rates to AEDs are ∼72 to 76%.^1^ New effective and noninvasive treatments for AED-refractory pediatric epilepsy are critically needed. Transcranial photobiomodulation (tPBM) with near-infrared (NIR) light refers to applying NIR transcranially according to the principle of photobiomodulation (PBM, previously termed low-level laser therapy).^2^ The mechanism of tPBM is based on photon absorption by mitochondrial cytochrome c oxidase (CCO).^3^ PBM has been studied in neurological diseases for 30 years.^4^ We recently demonstrated that tPBM (808 nm) attenuates seizures and status epilepticus (SE) induced by pentylenetetrazole (PTZ) in peripubertal rats.^5^ The behavioral and histopathological results showed that the treatment accomplished this (i) by reducing neuronal damage in the cortex, hippocampus (both GABAergic interneurons and principal cells), thalamus, and hypothalamus; (ii) by protecting hippocampal parvalbumin-positive interneurons—GABAergic interneurons that powerfully inhibit principal cells but are vulnerable to SE-induced apoptosis—from SE-induced apoptosis;^6^ and (iii) by preserving the integrity of the PV-immunoreactive perisomatic neuronal network surrounding the principal cells.^7^ Vogel et al.^8^ further revealed that repetitive tPBM (780 nm) reduced epileptiform discharges in a rat model of stroke-induced epilepsy. Regarding neuroprotection, tPBM has shown effects in reducing neuroinflammatory cytokines,^7^^,^^9^ which along with neuron-specific enolase (NSE) increased in the hippocampus after convulsive status epilepticus (CSE).^10^^,^^11^

The effects of tPBM in epilepsy on cerebral cell types other than neurons, such as glia (including astrocytes) and microglia, are poorly understood. Neuroinflammation plays critical roles in childhood epilepsy.^12^ It is frequently activated in certain brain regions involved in epileptogenesis^13^ and leads to focal astrogliosis and microgliosis of the lesions.^14^ Previous studies have shown that PBM reduces astrogliosis and microgliosis in the basal ganglia of aged mice,^15^ and also reduces astrogliosis in the midbrain and striatum in mouse^16^ and monkey^17^ models of Parkinson’s disease. Further, tPBM reduces levels of glial fibrillary acidic protein (GFAP), a cell marker for astrocytes and astrogliosis, in the subventricular zone in mice with traumatic brain injury^18^ and the ischemic cortex in mice with focal cerebral ischemia.^19^ Treatment with PBM also reduced neuroinflammation and microgliosis in the cortex in a mouse model of stroke^7^ and microgliosis in a mouse model of Alzheimer’s disease.^20^ However, whether tPBM reduces astrogliosis or microgliosis in the epileptic brain has yet to be examined directly. Relevantly, Vogel et al.^21^ reported that tPBM reduced the increased numbers of microglia present in the perilegional brain region after photothrombosis-induced ischemic stroke. The same animal model was also used by Vogel et al.^8^ to evaluate stroke-induced epilepsy.

The hippocampus is considered to play a critical role in epileptogenesis, and it is consistently activated during CSE.^22^ Levels of immunoreactivity of NSE—a biomarker of neuronal inflammation and injury—increased in the hippocampus of rats following PTZ-induced generalized tonic–clonic seizures or CSE.^11^ Levels of NSE increased not only in the epileptic brain parenchyma, but also in the cerebrospinal fluid^23^ and serum.^24^ PBM reduced serum NSE levels in patients with diabetic peripheral neuropathy.^25^ However, whether PBM reduces NSE immunoreactivity in the hippocampus of rats with seizures or SE is not known.

Here, we examine the effects of tPBM (808 nm) on neuroinflammation caused by PTZ-induced epilepsy and gliosis, including astrogliosis and microgliosis, in the hippocampus. We hypothesize that tPBM (808 nm) attenuates neuroinflammation, astrogliosis, and microgliosis in peripubertal rats with PTZ-induced SE.

## 2 Materials and Methods

### 2.1 Animals

The use of animals in this study complied with animal research guidelines for reporting of *in vivo* experiments and the Basel Declaration with consideration of the 3R principles. The study was approved by the Laboratory Animal Center of Taipei Medical University (No. LAC-2019-0237). Postnatal day 30 to 36 rats were purchased from BioLASCO (Taiwan). For this study, we performed brain sections from two pairs (behavioral analysis of a rat in PTZ and tPBM + PTZ groups, respectively, were assigned as a pair) of Sprague–Dawley rats from our previous study.^5^ Immunofluorescence staining of all markers in brain sections with N=2 animals were conducted, and N=1 animal with the most prominent therapeutic effects of tPBM on seizure behavior was designated for quantitative analysis of GFAP and ionized calcium-binding adapter molecule 1 (Iba-1) and characteristic analysis of mitochondrial cytochrome c oxidase subunit 1 (MT-CO1). These peripubertal rats were taken from groups treated with PTZ (rats PTZ1 and PTZ7, seizure behavioral experiments, and transcardial perfusion at postnatal day 30, respectively) or with both tPBM and PTZ (rats tPBM + PTZ1, seizure behavioral experiment at postnatal day 30 and transcardial perfusion at postnatal day 37, and tPBM + PTZ7 seizure behavioral experiment at postnatal day 30 and transcardial perfusion at postnatal day 64) in which the anticonvulsive effect of tPBM was second strongest in tPBM + PTZ1 and strongest in tPBM + PTZ7. For the control group, we analyzed brain sections of rats from our previous study. For this analysis, we examined the brain section of one rat from the saline-only group and the brain section of one rat from the tPBM group.

### 2.2 tPBM

We used a gallium aluminum arsenide (GaAlAs) diode laser apparatus (Transverse Industries, Taiwan) that we have described earlier.^5^^,^^26^ The center wavelength was 808 nm, and average radiant power was 110 mW per laser, and the apparatus was operated in continuous mode. The lens hood, which is a ring-shaped surface, is at a height of 11 mm from light source, and the rat scalp attached to the surface. The GaAlAs diode laser formed a central light beam and surrounding halos under the effects of the lens hood. The central light beam was in an elliptical shape, with major and minor axes measuring 3.5 and 3 mm, respectively, yielding an irradiated area of $0.0825\;{\mathrm{cm}}^{2}$. The irradiance at the scalp surface was approximately $1.333\;W/{\mathrm{cm}}^{2}$. The duration of exposure to the tPBM was 100 s, yielding a radiant exposure of approximately $133.3\;J/{\mathrm{cm}}^{2}$ and total radiant energy of 11 J per rat. The tPBM treatment was performed prior to administration of the PTZ injection.^5^

### 2.3 Acute Seizure Induction

A single dose of 90  mg/kg PTZ was subcutaneously injected into the rats in the PTZ and tPBM + PTZ groups. Normal saline was subcutaneously injected into the rats in the saline and the tPBM + saline groups.^5^

### 2.4 Transcardial Perfusion and Brain-Section Preparation

Rats that died from SE (PTZ1 and PTZ7) received transcardial perfusion^27^ with 4% paraformaldehyde immediately after death. Rats that survived the SE received transcardial perfusion a week (tPBM + PTZ1) or a month (tPBM + PTZ7) after the PTZ injection. Following the transcardial perfusion, the rats’ brains were harvested and stored in 20% sucrose overnight and then moved to and preserved in 30% sucrose at 4°C until use. For section preparation, the brains were immersed in the Tissue-Tek^®^ O.C.T.™ compound and then frozen in a bath of 2-methylbutane (M32631-1L, Sigma–Aldrich) and then soaked in liquid nitrogen for cooling. The OCT-embedded brains were stored at −20°C. Brain sections were cut with a thickness of 10  μm using a cryostat microtome (CM3050S, Leica, Germany) with settings of chamber temperature=−25°C and object temperature=−25°C^5^ and preserved at −80°C until use.

### 2.5 Immunofluorescence Staining

Immunofluorescence staining for NSE was mouse γ enolase (NSE-P1) monoclonal antibody (1:500; sc-21738, SANTA CRUZ); immunofluorescence staining with rabbit polyclonal GFAP antibody (with ratio either 1:200 or 1:400; GTX108711, and GTX27260 GeneTex) was performed to assess astrogliosis. Immunofluorescence staining with rabbit polyclonal Iba-1, (product full name with allograft inflammatory factor 1, synonyms as ionized calcium-binding adapter molecule 1 antibody) antibody (1:200, GTX101495, GeneTex) was performed to assess microgliosis. The secondary antibody for GFAP and Iba-1 was anti-rabbit IgG-fluorescein isothiocyanate (FITC) antibody produced in goat (1:600; F9887, Sigma–Aldrich). Immunofluorescence staining of MT-CO1 was performed using rabbit monoclonal anti-MT-CO1 antibody (1:1000; ab203917, Abcam) following Purushothuman et al.^28^ and the secondary antibody was Cy3-AffiniPure donkey anti-mouse IgG (H+L; 715-165-151, Jackson ImmunoResearch).

### 2.6 Quantitative Analysis of NSE-Immunoreactive Neurons

Brain sections from the rats in which tPBM had the second-strongest anticonvulsive effect (tPBM + PTZ1) and the strongest anticonvulsive effect (tPBM + PTZ7) and their pairs (PTZ1 and PTZ7, the pairs were based on the matched body weight of rats with matched PTZ injection volume) in our previous study^5^ were selected for analysis of neuroinflammation using the marker NSE. The secondary antibody used for PTZ1 and tPBM+PTZ1 was Cy3-AffiniPure donkey anti-mouse IgG and for PTZ7 and tPBM+PTZ7 was anti-mouse IgG-FITC antibody. We used two different secondary antibodies to minimize interference from background signal in the brain parenchyma as shown by Song et al.^29^ Enlargement of NSE-immunoreactive (NSE-IR) and immunonegative area with magnitude of 900% was performed by Microsoft PowerPoint.

### 2.7 NSE and GFAP Double Immunofluorescence Staining

To determine neuronal damage and astrogliosis, we performed double immunofluorescence staining of NSE and GFAP. The primary antibody of NSE was mouse γ enolase (NSE-P1) monoclonal antibody (1:500; sc-21738, SANTA CRUZ) as mentioned earlier. The primary antibody of GFAP was Rabbit polyclonal anti-GFAP antibody (GTX27260, GeneTex). Images of the sections were obtained using an Observer.Z1 microscope fitted with an Axiocam ERc 5s camera (ZEISS). We adjusted the images of NSE and GFAP using the “best-fit” function in the ZEN software package ZEN blue version 3.3.89.0000 (Carl Zeiss Microscopy GmbH) with the settings of “black” as 2% for 4’,6-diamidino-2-phenylindole and 95% for FITC (corresponding to GFAP) and cyanine 3 (Cy3, corresponding to NSE). We analyzed the cell area of 20 astrocytes in each microscope field at 200× magnification in each group to approximate all astrocyte in each field to minimize selection bias. To compare cell area in four groups with the same condition, we analyze identical cell number in each group. The number “20” was close to the astrocyte number in saline group, which was the least among four groups (the cell number of tPBM + saline group was near that in saline group). Cell area was obtained by freehand selection of the contour of astrocytes by ImageJ software [National Institutes of Health (NIH)]. Considering that the cell number of microglia was close to 10 microglia in each microscope field at 200× magnification, we analyze the cell area of 10 microglia in each field accordingly.

### 2.8 Imaging Processing of GFAP, Iba-1, and MT-CO1 Immunofluorescence

After taking images from the microscope, the FITC channel of GFAP remained as green as default, and the FITC channel of Iba-1 changed to yellow. Color images of GFAP, Iba-1, and MT-CO1 immunofluorescence were first inverted and then adjusted to black and white with maximum black in Adobe Photoshop CS5 (64 bit). Next, to analyze the morphology of the microglia, the area of interest was cropped to a square area with size of 0.5×0.5 (150×150  pixels) and the contrast was enhanced by 3% in ImageJ software (NIH). Enlargement of MT-CO1 IR area with magnitude of 720% was performed by Microsoft PowerPoint.

### 2.9 Statistical Analysis

One-way analysis of variance (ANOVA) with *post hoc* Tukey’s multiple comparisons was used to analyze the NSE-IR neurons, GFAP-IR astrocytes, and Iba-1-IR microglia.

## 3 Results

### 3.1 tPBM Significantly Reduced NSE-IR Neurons in the CA3 Region

**Fig. 1:**
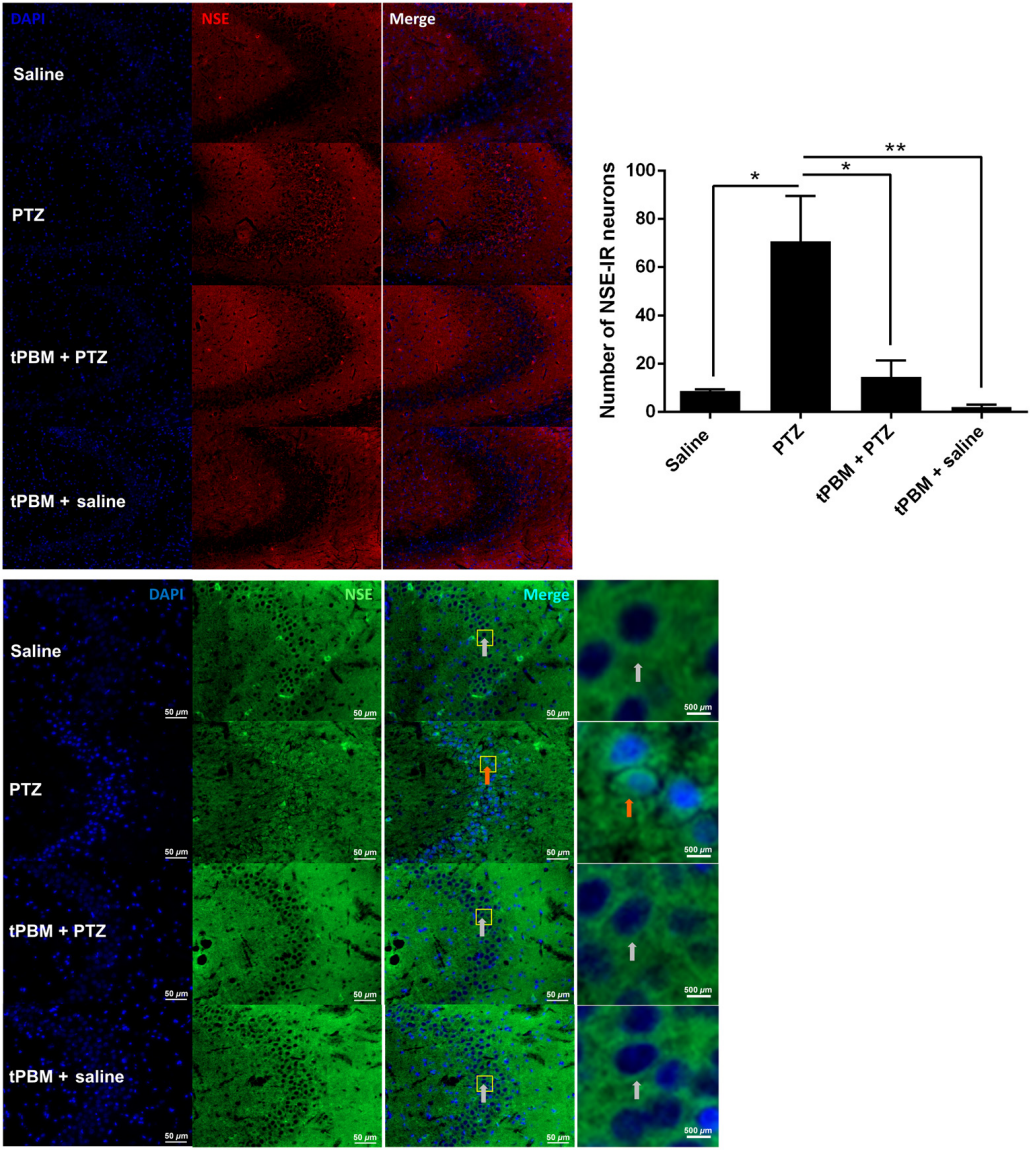
Quantitative analysis of effects of tPBM on NSE-IR neurons in the CA3 region. Brain sections from rats were analyzed for neuroinflammation using the marker NSE, with secondary antibodies for Cy3 (red) and FITC (green) (scale bar=50  μm). Data derived from images with Cy3 and FITC were pooled for analysis (n=2 animals from the PTZ and tPBM + PTZ groups and one each from the saline and tPBM + saline groups, * p<0.05, ** p<0.01). Orange arrows indicate representative NSE-IR neurons in the PTZ group, and gray arrows indicate corresponding NSE-immunonegative neurons in CA3. Subfigures were enlarged from merged images (scale bar=500  μm).

There were significant differences in all groups (p=0.0058, one-way ANOVA), and the NSE in the tPBM + PTZ group was significantly less IR than that in the PTZ group. Specifically, in the PTZ group, the number of NSE-IR neurons (70.75±18.78) was significantly greater than in the saline group (8.67±0.67;p<0.05) and the tPBM + PTZ group (14.50±6.90; p<0.05, *post hoc* multiple comparison; Fig. 1 and Table 1).

**Table 1:** The effects of tPBM on PTZ-induced neuroinflammation in the hippocampus.

	Saline	PTZ	tPBM + PTZ	tPBM + saline
Number of NSE-IR neurons	8.67 ± 0.67	70.75 ± 18.78	14.50 ± 6.90	2.00 ± 1.16

Number of NSE-IR neurons in the CA3 region per microscope field at 200× magnification. Data are expressed as mean ± standard error of the mean (SEM). PTZ, pentylenetetrazole; tPBM, transcranial photobiomodulation; NSE, neuron-specific enolase.

### 3.2 tPBM Reduced Neuroinflammation and Astrogliosis in the CA3 Region

**Fig. 2:**
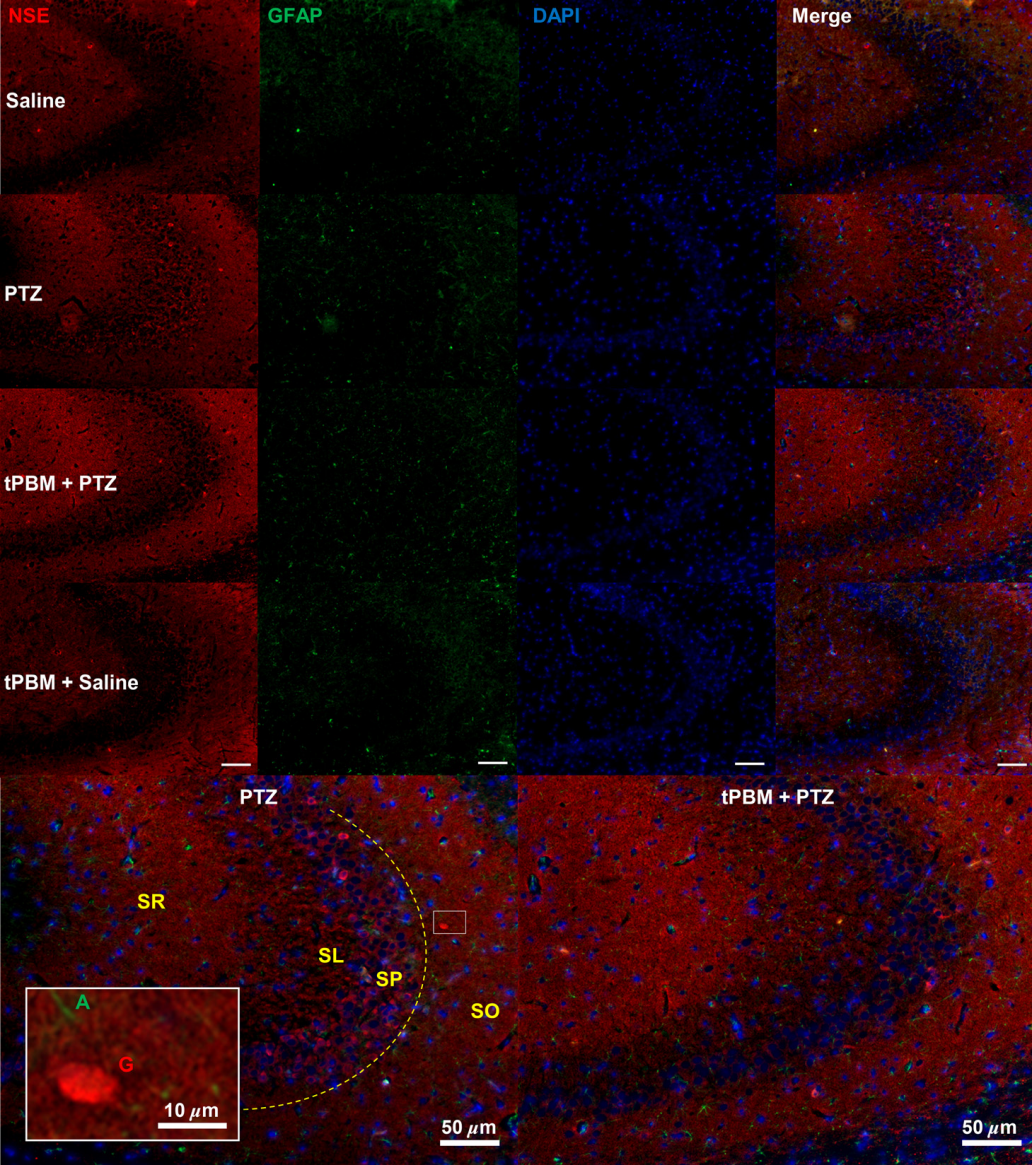
The effects of tPBM on PTZ-induced neuroinflammation and astrogliosis in the CA3 region. Double immunofluorescence staining of NSE (red) and GFAP (green) in the CA3 illustrates the effect of tPBM on neuroinflammation (indicated by NSE) and astrogliosis (GFAP) and their combination. NSE was stained in the cytosol of inflammatory neurons. Pyramidal neurons were aligned at SP. Neurons located at other layer such as at SO were GABAergic interneurons. Astrocytes were stained for GFAP, and an increase in GFAP immunoreactivity, somata size, and branching indicated astrogliosis. DAPI (blue) was used as a counterstain for the nucleus (scale bar=50  μm). The two images in the lower panel are enlargements of the merged images from the PTZ and tPBM + PTZ groups, respectively. Enlargement: a GABAergic interneuron (labeled “G”) based on the morphology with smaller nuclear–cytoplasmic ratio compared with that of principle cells; the process of an astrocyte (A) was adjacent to the GABAergic interneuron (scale bar=10  μm). Stratum lucidum, SL; stratum radiatum, SR.

Double immunofluorescence staining for NSE and GFAP revealed that tPBM (808 nm) reduced both NSE-IR neuron numbers and the immunoreactivity of GFAP-IR astrocytes in the CA3 (CA = cornus ammonis) region of the hippocampus. This indicates that tPBM suppressed neuroinflammation and astrogliosis simultaneously. NSE, a biomarker of neuroinflammation, is expressed in the cytosol of neurons. Rats in the saline group had few NSE-positive neurons. However, many NSE-positive neurons were observed in the CA3 of rats in the PTZ group, but there were far fewer NSE-IR cells in the tPBM + PTZ group (Fig. 2). Both excitatory pyramidal neurons and GABAergic inhibitory interneurons were NSE-IR in the CA3 region in the PTZ group, indicating neuroinflammation in both the GABAergic inhibitory interneurons and excitatory principal cells in that region.

### 3.3 tPBM Attenuated Astrogliosis in the Hippocampus and White Matter in Rats with PTZ-Induced SE

**Fig. 3:**
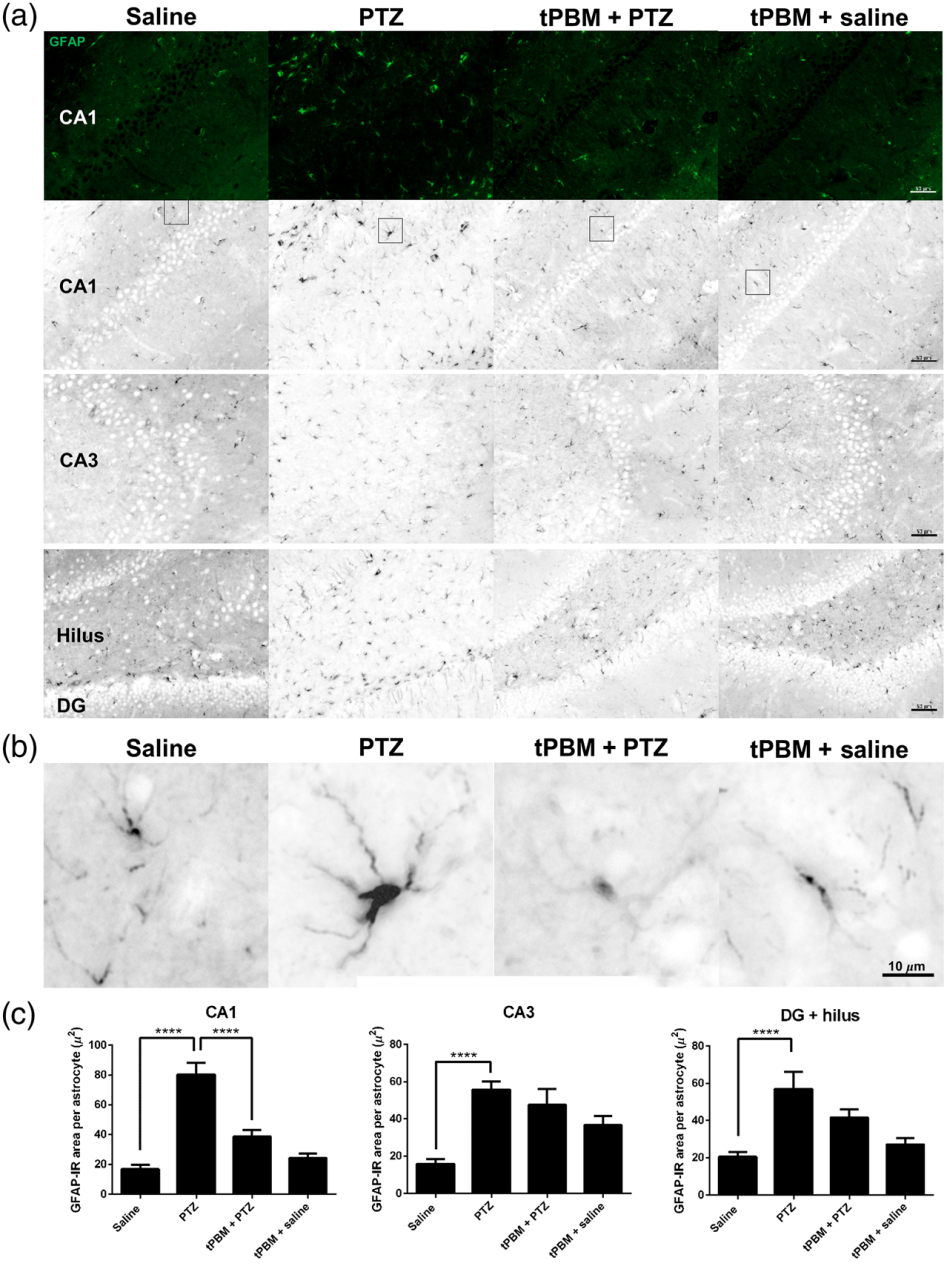
Effects of tPBM on astrogliosis. (a) Astrocytes stained for GFAP are shown in black. Astrocytes with enlarged somata and branched processes indicate astrogliosis. Astrocytes in all four treatment groups (saline, PTZ, tPBM + PTZ, and tPBM + saline) in the CA1, CA2, CA3, hilus and DG regions are displayed (scale bar=50  μm). (b) Representative astrocytes selected from SO in CA1 region; images were enlarged from the square area in (a) (scale bar=10  μm). (c) Statistical results. One-way ANOVA. ***Adjusted p<0.0001.

Baseline GFAP-IR astrocytes were observed in the CA1, CA2, CA3, and dentate gyrus (DG) (including the hilus) regions of the hippocampus. Astrogliosis, represented by prominent GFAP-IR somata and branches, was observed in the PTZ group. The astrocyte somata were smaller and the cells had fewer, shorter branches in the tPBM + PTZ group, suggesting that tPBM suppressed astrogliosis in rats with PTZ-induced SE [Fig. 3(a)]. Representative enlarged images of astrocyte including the manifestation of astrogliosis in PTZ group were shown [Fig. 3(b)]. Further quantitative analysis of GFAP IR cell area of the astrocyte in CA1 showed that the cell area in PTZ group ($80.55\pm 7.69\;\mu^{2}\;\mathrm{per}\;\text{cell}$) was significantly larger compared to that in saline group ($17.03\pm 2.84\;\mu^{2}\;\mathrm{per}\;\text{cell}$) (adjusted p<0.0001), whereas the astrocytic cell area in tPBM + PTZ group ($38.64\pm 4.51\;\mu^{2}\;\mathrm{per}\;\text{cell}$) was significantly reduced compared with those in PTZ group (adjusted p<0.0001. Fig. 3(c)]. As for the astrocytic area in CA3, there was a significant increase in the cell area of the astrocyte in PTZ group ($55.89\pm 4.30\;\mu^{2}\;\mathrm{per}\;\text{cell}$) compared with those in saline group ($15.87\pm 2.58\;\mu^{2}\;\mathrm{per}\;\text{cell}$). There was no significant difference between the cell area of astrocytes in tPBM + PTZ group ($47.60\pm 8.48\;\mu^{2}\;\mathrm{per}\;\text{cell}$) and those in PTZ group. Similar trend of cell area in DG and hilus was noted. The size of astrocyte in DG and hilus in saline group was ($20.56\pm 2.58\;\mu^{2}\;\mathrm{per}\;\text{cell}$), and the cell size was significantly larger in PTZ group ($57.10\pm 9.35\;\mu^{2}\;\mathrm{per}\;\text{cell}$, adjusted p<0.0001), whereas the astrocyte size in DG and hilus in tPBM + PTZ group (41.77±4.19) and that in PTZ group was not significantly different [Fig. 3(c); Table 2].

Next, we evaluate the protective effect of tPBM against astrogliosis in the white matter surrounding the hippocampus (Fig. S1 in the Supplementary Material). We observed more prominent astrogliosis in the white matter (including the cingulum, alveus hippocampus, fimbria hippocampus, and lateral ventricle) surrounding the hippocampus than in the hippocampus *per se* in the PTZ group. Nevertheless, no obvious astrogliosis in the peri-hippocampal white matter was detected in the saline or tPBM + PTZ groups (Fig. S1 in the Supplementary Material). These results indicate that tPBM suppressed astrogliosis in both the hippocampus and the peri-hippocampal white matter as well.

**Table 2:** The effects of tPBM on PTZ-induced astrogliosis in the hippocampus.

Subregion	GFAP-IR cell area per astrocyte ($\mu^{2}$)			
	Saline	PTZ	tPBM + PTZ	tPBM + saline
CA1	17.03 ± 2.84	80.55 ± 7.69	38.64 ± 4.51	24.32 ± 2.83
CA3	15.87 ± 2.58	55.89 ± 4.30	47.60 ± 8.48	36.71 ± 4.84
DG + hilus	20.56 ± 2.58	57.10 ± 9.35	41.77 ± 4.19	27.25 ± 3.25

Twenty astrocytes per microscope field at 200× magnification in the CA1, CA3, and DG and hilus subregion were selected for analysis. Data are expressed as mean ± SEM. PTZ, pentylenetetrazole; tPBM, transcranial photobiomodulation; GFAP, glial fibrillary acidic protein.

### 3.4 tPBM Attenuates Microgliosis in the Hippocampus in Rats with PTZ-Induced SE

**Fig. 4:**
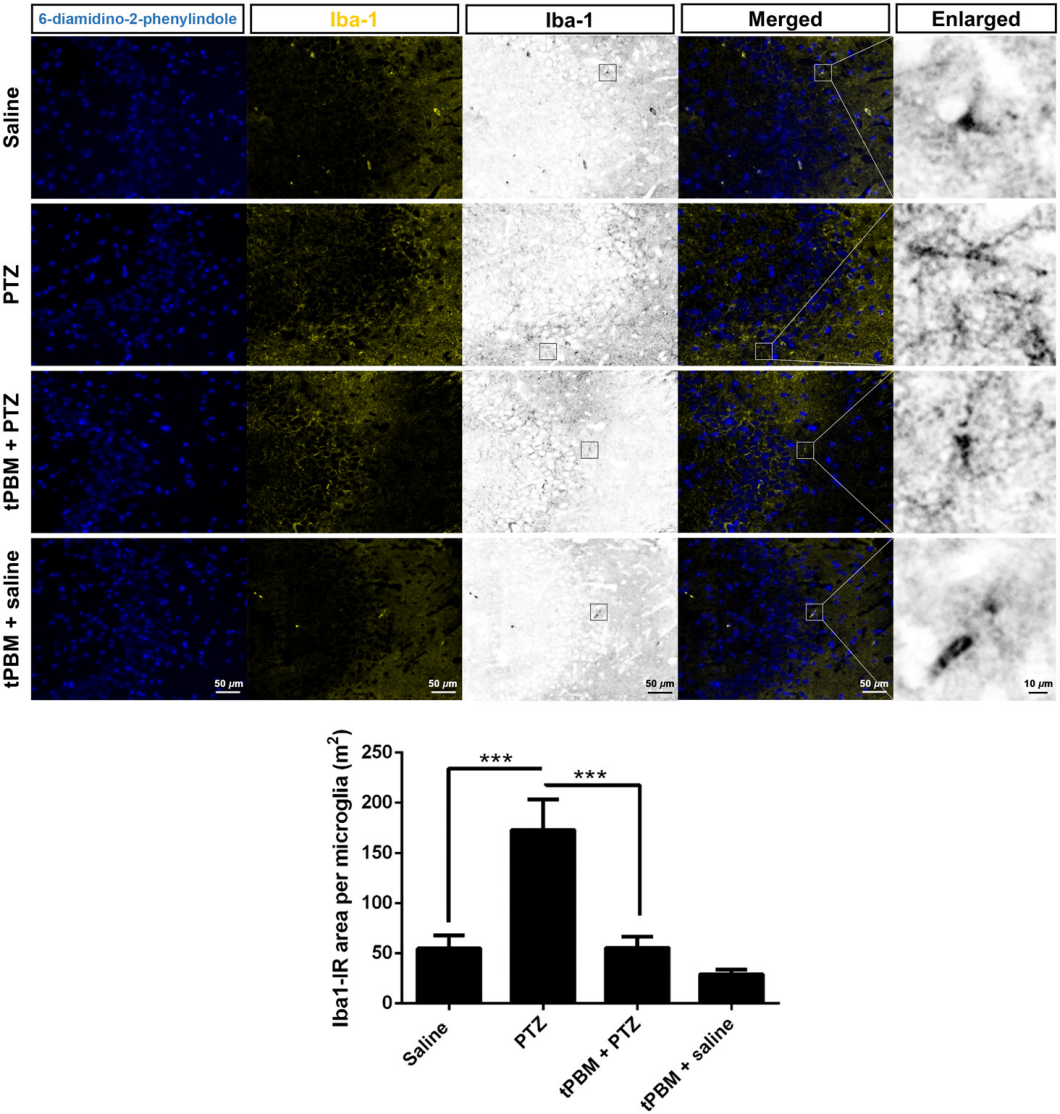
Effects of tPBM on microgliosis in CA3. Iba-1 immunoreactive microglia were shown in yellow (scale bar=50  μm). Representative microglia (enlarged and black-and-white processing) with little branching are in the resting state and exhibit a ramified morphology. Microglia after SE presented with much branching are 545 in the reactive state (microgliosis) and exhibit a hyperramified (hypertrophic) and bushy morphology (scale bar=10  μm). Quantitative analysis of the Iba-1 immunoreactive microglia cell area is shown below. One-way ANOVA. ***Adjusted p=0.0002.

The Iba-1-IR microglia exhibited a ramified morphology in the CA3 in the saline group. Microgliosis with microglia, which presents as bushy morphology, was noted in the stratum oriens (SO) of the CA3 region in the PTZ group. Nevertheless, the microglia in the SO in tPBM + PTZ group were less branched than those in the PTZ group (Fig. 4). Quantitative analysis of Iba-1 IR microglial cell area revealed significantly increased cell area in PTZ group ($172.9\pm 30.35\;\mu^{2}\;\mathrm{per}\;\text{cell}$) compared with those in saline group ($54.97\pm 12.90\;\mu^{2}\;\mathrm{per}\;\text{cell}$, adjusted p=0.0002), and the microglial cell area in the tPBM + PTZ group ($55.58\pm 11.14\;\mu^{2}\;\mathrm{per}\;\text{cell}$) was significantly reduced compared with those in the PTZ group (adjusted p=0.0002; Fig. 4; Table 3).

**Table 3:** The effects of tPBM on PTZ-induced microgliosis in the hippocampus.

Subregion	Iba-1-IR cell area per astrocyte ($\mu^{2}$)			
	Saline	PTZ	tPBM + PTZ	tPBM + saline
CA3	54.97 ± 12.90	172.9 ± 30.35	55.58 ± 11.14	29.19 ± 4.72

Ten microglia per microscope field at 200× magnification in the CA3 subregion were selected for analysis. Data are expressed as mean ± SEM. PTZ, pentylenetetrazole; tPBM, transcranial photobiomodulation; Iba-1, ionized calcium-binding adaptor molecule 1.

### 3.5 tPBM Increased the Immunoreactivity of MT-CO1

**Fig. 5:**
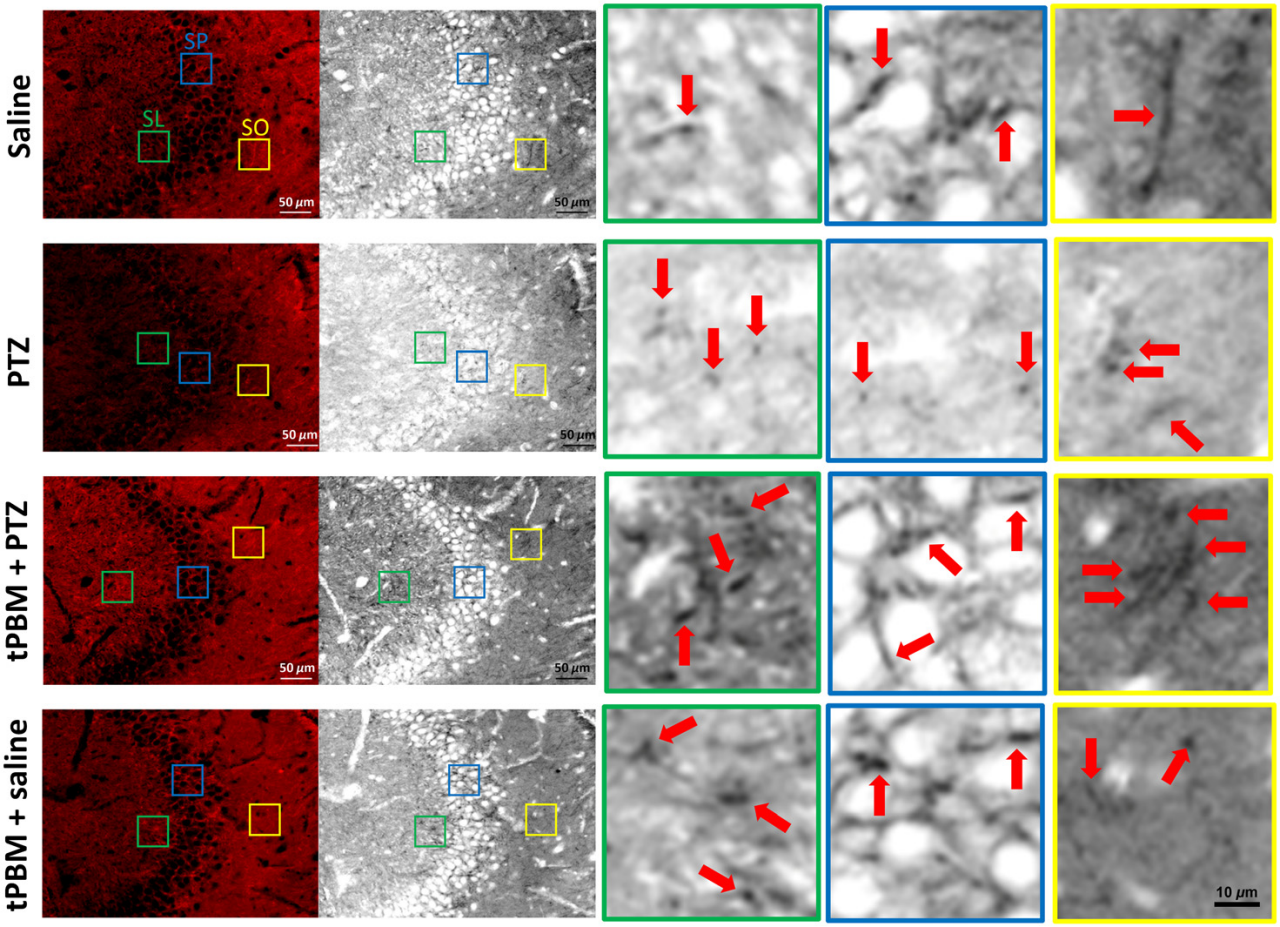
MT-CO1 immunoreactivity in the CA3 region. MT-CO1 immunofluorescence staining images (left) and their black-and-white processing counterparts (middle) for the four treatment groups (saline, PTZ, tPBM + PTZ, and tPBM + saline) were displayed (scale bar=50  μm). SO, stratum oriens; SP, stratum pyramidale; SL, stratum lucidum. The 720% enlargement of length and width of the indicated green, blue, and yellow squares were representative subfigures of MT-CO1 immunoreactive cells (red arrows pointed to some but not all MT-CO1 immunoreactive cells) in SL, SP, and SO, respectively (scale bar=10  μm).

Finally, to confirm that the protective effect was elicited directly by tPBM, we performed immunofluorescence staining of MT-CO1. Here, we took CA3 as the subregion of interest. We found that immunoreactivity in the CA3 region in the tPBM + PTZ group was greater than that in the PTZ group. Notably, the MT-CO1-IR cells were situated next to pyramidal neurons. Specifically, the immunoreactivity of the MT-CO1 cells was enhanced in the stratum lucidum (SL) layer of the CA3 in the tPBM + saline group relative to the saline group. Moreover, the MT-CO1 immunoreactivity of the SL in the tPBM + PTZ group was higher than in the PTZ group (Fig. 5). Further identification of the MT-CO1 IR part of the cells revealed that the MT-CO1 immunoreactivity was identical to neurite (either dendrite or axons) or cytosol. As for MT-CO1 IR cells among the layers in CA3, columnar-shaped MT-CO1 IR cells were presented richly in SL layer in the tPBM + PTZ group and the tPBM + saline group compared with that in SL layer of the PTZ group and saline group. Regarding the MT-CO1 immunoreactivity of SP layer, the MT-CO1 IR neurite was observed to surround the halo of pyramidal cells without exception. However, the MT-CO1 immunoreactivity of neurite in SP was poor in the PTZ group. Interestingly, spindle-shaped elongated Iba-1 IR cells were noted at SO in saline group, and they were not prominent in the PTZ group. Similar morphology of cells was presented in SO layer of the tPBM + PTZ group (Fig. 5).

## 4 Discussion

In this study, we demonstrated that tPBM (808 nm) suppressed neuroinflammation, astrogliosis, and microgliosis in hippocampus of peripubertal rats with PTZ-induced SE in a CCO-dependent manner. SE induced by PTZ caused neuroinflammation in both GABAergic interneurons and pyramidal neurons in the CA3 region, which indicates that both the inhibitory GABAergic interneurons and the excitatory principal cells are involved in PTZ-induced or SE-induced neuroinflammation. In contrast, tPBM suppressed the neuroinflammation of the GABAergic interneurons in the CA3. These findings were consistent with those of our previous study,^5^ in which both hippocampal GABAergic interneurons and principal cells appeared dark under hematoxylin and eosin staining in the PTZ group, whereas there were only a few dark neurons in the tPBM-treated rats.^5^

With respect to astrogliosis, tPBM reduced astrogliosis, and this was consistent with previous studies.^17^^,^^30^^,^^31^ Deregulation of ATP signaling in astrocytes and the release of ATP (extracellular ATP acts as a purinergic signal) from astrocytes during astrogliosis is known to contribute to epileptogenesis by lowering the seizure threshold of excitatory neurons.^32^ Regarding the effect of PBM on purinergic signaling, de Freitas and Hamblin^33^ stated in 2016, “Up to the present date we are not aware of any studies that specifically show that extracellular (as opposed to intracellular) ATP or adenosine can be stimulated by PBM.”^33^ We propose that, instead of stimulating extracellular ATP or adenosine, tPBM may attenuate astrogliosis-related purinergic signaling in epilepsy in three ways. First, tPBM may reduce the release of ATP from astrocytes by preventing the astrocytes from developing astrogliosis during seizures. Second, tPBM may facilitate the hydrolyzation of ATP into adenosine and increase adenosine 1 receptor expression in excitatory principal cells so as to promote the anticonvulsant effects of adenosine.^34^ Third, tPBM may reduce ATP-gated P2X7 receptor expression in the microglia in epilepsy since PBM reduced the expression of purinergic P2X7 receptor in lung tissue in a mouse model of chronic obstructive pulmonary disease.^35^ Furthermore, expression of the IL-1 receptor/toll-like receptor superfamily in reactive astrocytes^36^ is upregulated in epilepsy.^37^^–^^39^ It is possible that tPBM suppresses this upregulation in SE since PBM is capable of suppressing proinflammatory IL-1 β in human adipose-derived stem cells^40^ and of suppressing IL-1 β in the ischemic brain and toll-like receptor-2 level in the postischemic brain.^7^

This is the first report that tPBM (808 nm) attenuates microgliosis with bushy morphology after SE.^41^ The similar pattern of immunoreactivity in the MT-CO1-IR cells and processes (notably that dendritic and axonal mitochondria could be MT-CO1-IR) and the Iba-1-IR microglial processes in the SL of the CA3 hint at the possibility that tPBM may also attenuate SE by strengthening the neuroprotective functions of microglia, such as microglial surveillance.^42^ Further experimental evidence to support this argument is needed.

In this study, the sequential arrangement of tPBM administered before PTZ injection was due to the consideration of accurate localization of tPBM and safety issue of rats in “CSE mode.” When rats developed CSE, their neck would shake heavily. Forced immobilization of the head to conform the apparatus of tPBM in such CSE mode would cause fracture of neck. On the contrary, tPBM administered prior to PTZ injection enable accurate localization of the head corresponds to targeted regions of the brain under stable condition of rats without the concern of neck fractures. As for the clinical application, tPBM as mean of CSE prevention is promising. In fact, instant intervention prior to CSE events with US Food and Drug Administration-approved responsive neurostimulation (RNS) system (NeuroPace) had been applied to patients with medically intractable focal onset seizures, and the RNS indeed reduced focal seizures and improved quality of life.^43^ According to our previous study^5^ and current study, tPBM might play roles in CSE prevention with combination of tPBM and mature seizure prediction.^44^ Regarding the dosage of tPBM, single dose was used in this study yet long-term beneficial effects were shown in histobiochemical studies. The choice of single-dose tPBM treatment session rather than repetitive treatment was due to the choice of acute seizure model elicit by PTZ and examination of effects of tPBM on acute seizures within short time windows (minutes in scale). As for the long-term beneficial effect, in a previous study,^45^ single PBM treatment session could cause long-term beneficial effects on brain injuries at tissue level. Considering that the dose of PTZ used in current study was a lethal dose, which would cause instant pathological insult on rat brains during CSE. Brains from rats died after SE were harvested immediately after death. Therefore, the state of “acute” neuroinflammation, astrogliosis, and microgliosis occurred. On the contrary, with the protection of tPBM, the brain insults were modest upon the observation period after PTZ injection. The neuroinflammation, astrogliosis, and microgliosis in tPBM + PTZ group at 14 and 30 days were modest accordingly. Taken together, the long-term beneficial effects of tPBM were contributed mainly by the protection of tPBM in the first hour after PTZ injection and such beneficial effects lasted.

### 4.1 Limitations

The photothermal effect of tPBM under irradiance of $1.333\;W/m^{2}$ was not measured in this study. This will be included in future studies on tPBM albeit the simulations of tPBM (810 nm) predict temperature increases of merely <0.25°C in the scalp and <0.04°C in the gray matter.^46^ In addition, insufficient rat number in saline and tPBM + saline group for blank control is the limitation for statistical rigor in this study.

## 5 Conclusions

tPBM with a wavelength of 808 nm suppressed PTZ-induced neuroinflammation, astrogliosis, and microgliosis in the hippocampus. It also enhanced CCO immunoreactivity in SL of CA3. These results may improve our understanding of the underlying antiepileptic mechanisms of tPBM, which should not be underestimated in future applications in pediatric epilepsy.
