# Supplementary material for: Transcranial photobiomodulation (808 nm) attenuates pentylenetetrazole-induced seizures by suppressing hippocampal neuroinflammation, astrogliosis, and microgliosis in peripubertal rats
Source: Neurophotonics. 2022 Mar 25;9(1):015006. doi: 10.1117/1.NPh.9.1.015006 (PMC8955735; doi:10.1117/1.NPh.9.1.015006)
Supplement: Supplementary file 1 [file NPh_009_015006_SD001.pdf]

Supplemental Material

Fig. S1 Effects of tPBM on astrogliosis in the white matter surrounding the hippocampus.

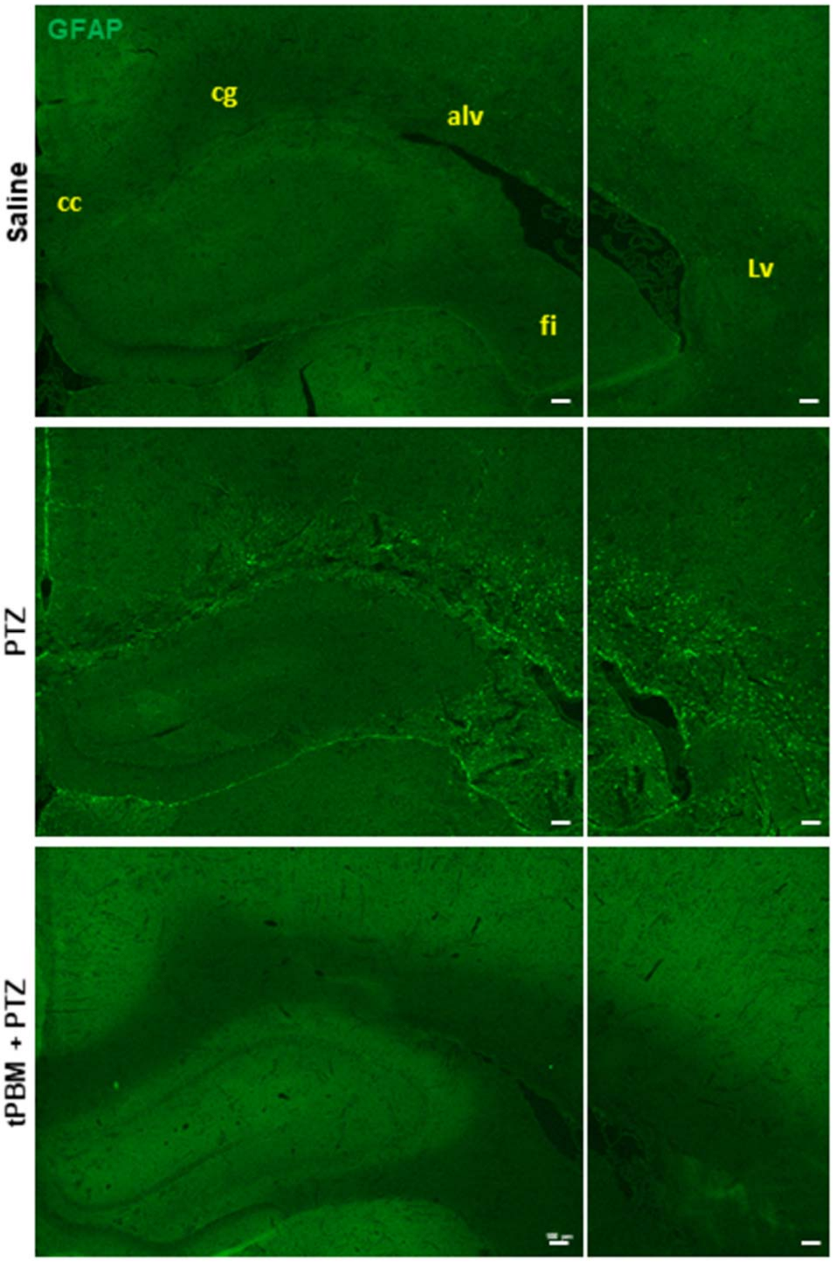

Fig. S1 Effects of tPBM on astrogliosis in the white matter surrounding the hippocampus. Effects of tPBM on astrogliosis in the white matter surrounding the hippocampus. Sections of hippocampi and surrounding white matter were stained for GFAP to reveal astrocytes. Astrocytes in the state of astrogliosis appear light green (scale bar = 100 μm). cc, corpus callosum; cg, cingulum; alv, alveus of the hippocampus; Lv, lateral ventricle; fi, fimbria of the hippocampus.
